# Supplementary figures and images for: Variable susceptibility of intestinal organoid–derived monolayers to SARS-CoV-2 infection
Source: PLoS Biol. 2022 Mar 31;20(3):e3001592. doi: 10.1371/journal.pbio.3001592 (PMC9004766; doi:10.1371/journal.pbio.3001592)

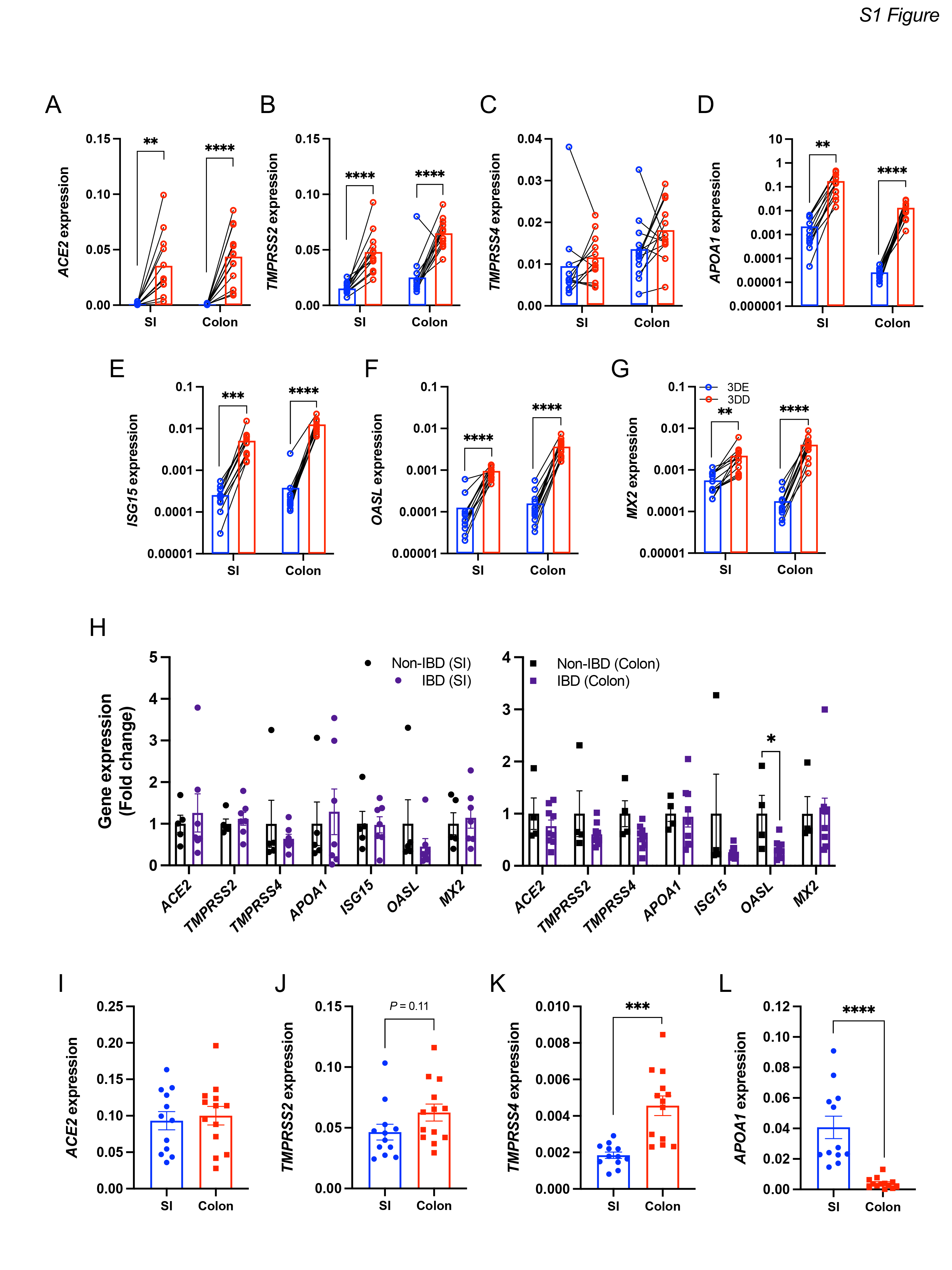

Supplement: S1 Fig — (A–G) RT-PCR analysis of ACE2 (A), TMPRSS2 (B), TMPRSS4 (C), APOA1 (D), ISG15 (E), OASL (F), and MX2 (G) expression among SI or colonic 3D organoids grown in expansion media (3DE) or differentiation media (3DD) for 7 days. (H) RT-PCR data showing ACE2, TMPRSS2, TMPRSS4, APOA, ISG15, OASL, and MX2 expression in 3DE organoids according to disease status. (I–L) RT-PCR analysis of ACE2 (I), TMPRSS2 (J), TMPRSS4 (K), and APOA1 (L) expression among SI and colonic monolayers grown in differentiation media for 7 days. Data points are mean of at least 2 technical replicates of individual organoid lines. Bars represent mean, and at least 2 independent experiments were performed. Underlying data can be found in S1 Data. P, P value. *P ≤ 0.05, **P ≤ 0.01, ***P ≤ 0.001, and ****P ≤ 0.0001 by paired t test, 2 tailed in A–G and unpaired t test, 2 tailed in H–L. ACE2, angiotensin I converting enzyme 2; ISG, interferon-stimulated gene; RT-PCR, reverse transcription PCR; SI, small intestine. (TIF) [file pbio.3001592.s002.tif]

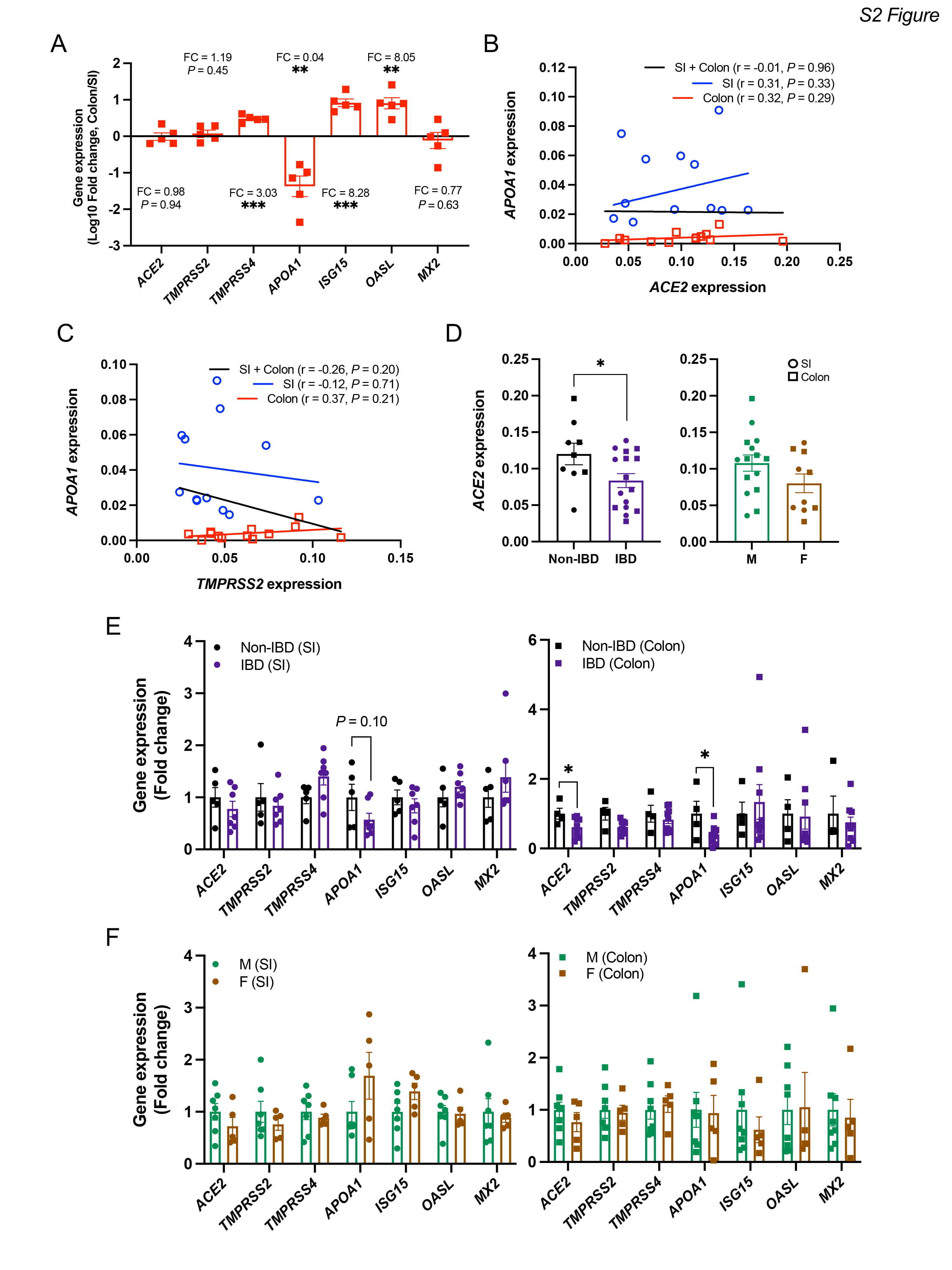

Supplement: S2 Fig — (A) RT-PCR analysis of ACE2, TMPRSS2, TMPRSS4, APOA1, ISG15, OASL, and MX2 expression between matched donor-derived SI and colonic monolayers grown in differentiation media for 7 days. Data are displayed fold-change differences between the expression of the indicated genes in colonic monolayers over expression in SI monolayers. (B and C) Correlation of ACE2 (B) and TMPRSS2 (C) expression with APOA1 expression among monolayers grown in differentiation media. (D) RT-PCR analysis of ACE2 expression among monolayers grown in differentiation media according to the disease status or sex of donors. (E and F) RT-PCR data depicting ACE2, TMPRSS2, TMPRSS4, APOA1, ISG15, OASL, and MX2 expression of monolayers according to disease (E) or sex (F). Data points are mean of at least 2 technical replicates of individual organoid lines. Bars represent mean ± SEM, and at least 2 independent experiments were performed. Underlying data can be found in S1 Data. FC, fold change; r, Pearson correlation coefficient; P, P value. *P ≤ 0.05, **P ≤ 0.01, and ***P ≤ 0.001 by paired t test, 2 tailed in A, simple regression analysis in B and C, and unpaired t test, 2 tailed in D–F. ACE2, angiotensin I converting enzyme 2; F, female; ISG, interferon-stimulated gene; M, male; RT-PCR, reverse transcription PCR; SI, small intestine. (TIF) [file pbio.3001592.s003.tif]

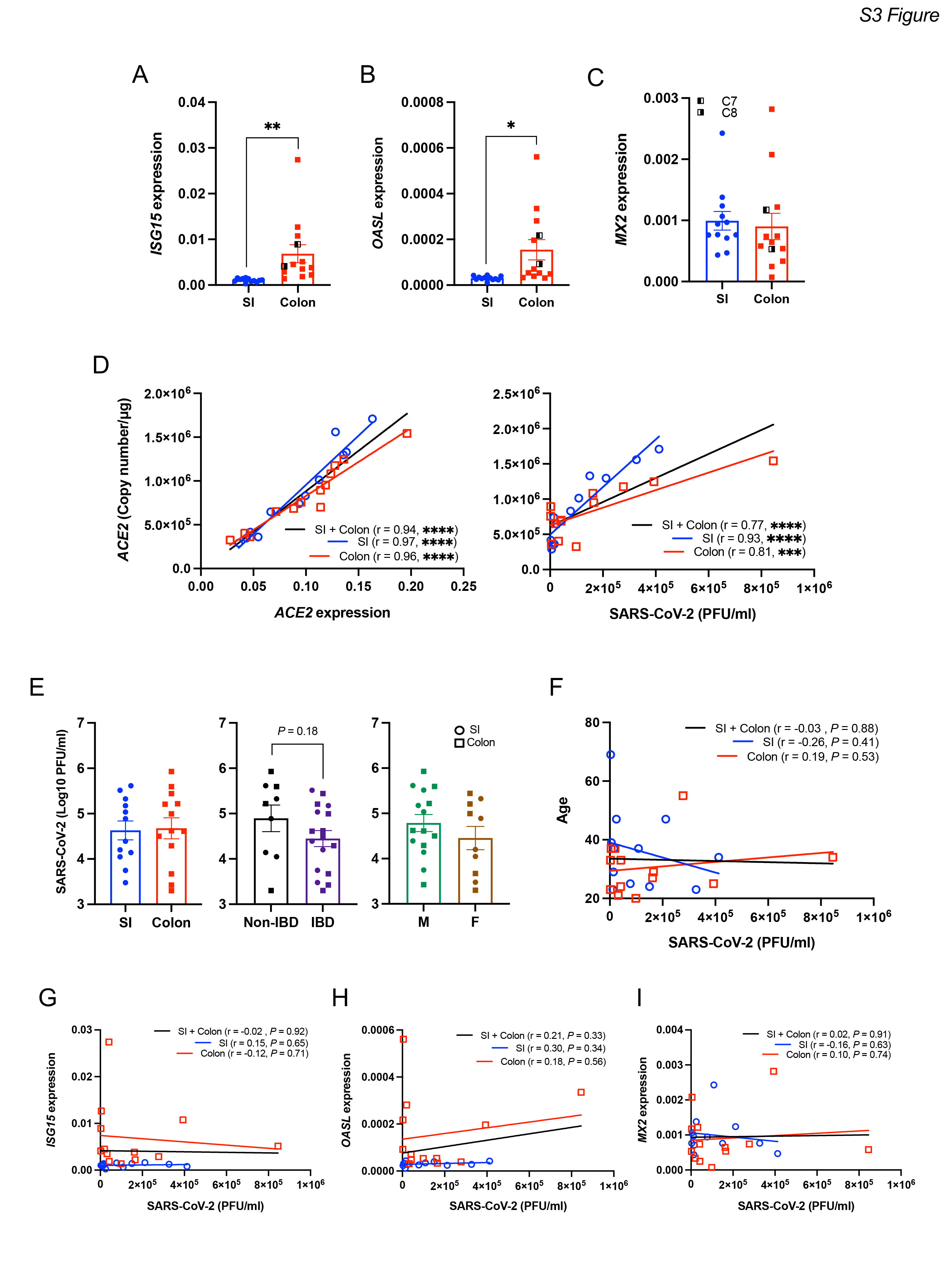

Supplement: S3 Fig — (A–C) RT-PCR analysis of ISG15 (A), OASL (B), and MX2 (C) expression in monolayers. (D) Correlation of ACE2 copy number with ACE2 expression (left) or PFU of SARS-CoV-2 (right). (E) PFU of SARS-CoV-2 according to intestinal region (left), disease (middle), or sex (right) of donors. (F–I) Correlation of PFU of SARS-CoV-2 with age of donors (F) or ISG15 (G), OASL (H), and MX2 (I) expression. Data points are mean of at least 2 technical replicates of individual organoid lines. Bars represent mean ± SEM, and at least 2 independent experiments were performed. Underlying data can be found in S1 Data. r, Pearson correlation coefficient; P, P value. *P ≤ 0.05, **P ≤ 0.01, ***P ≤ 0.001, and ****P ≤ 0.0001 by unpaired t test, 2 tailed in A–C and E and simple regression analysis in D and F–I. ACE2, angiotensin I converting enzyme 2; F, female; M, male; PFU, plaque-forming unit; RT-PCR, reverse transcription PCR; SARS-CoV-2, Severe Acute Respiratory Syndrome Coronavirus 2. (TIF) [file pbio.3001592.s004.tif]

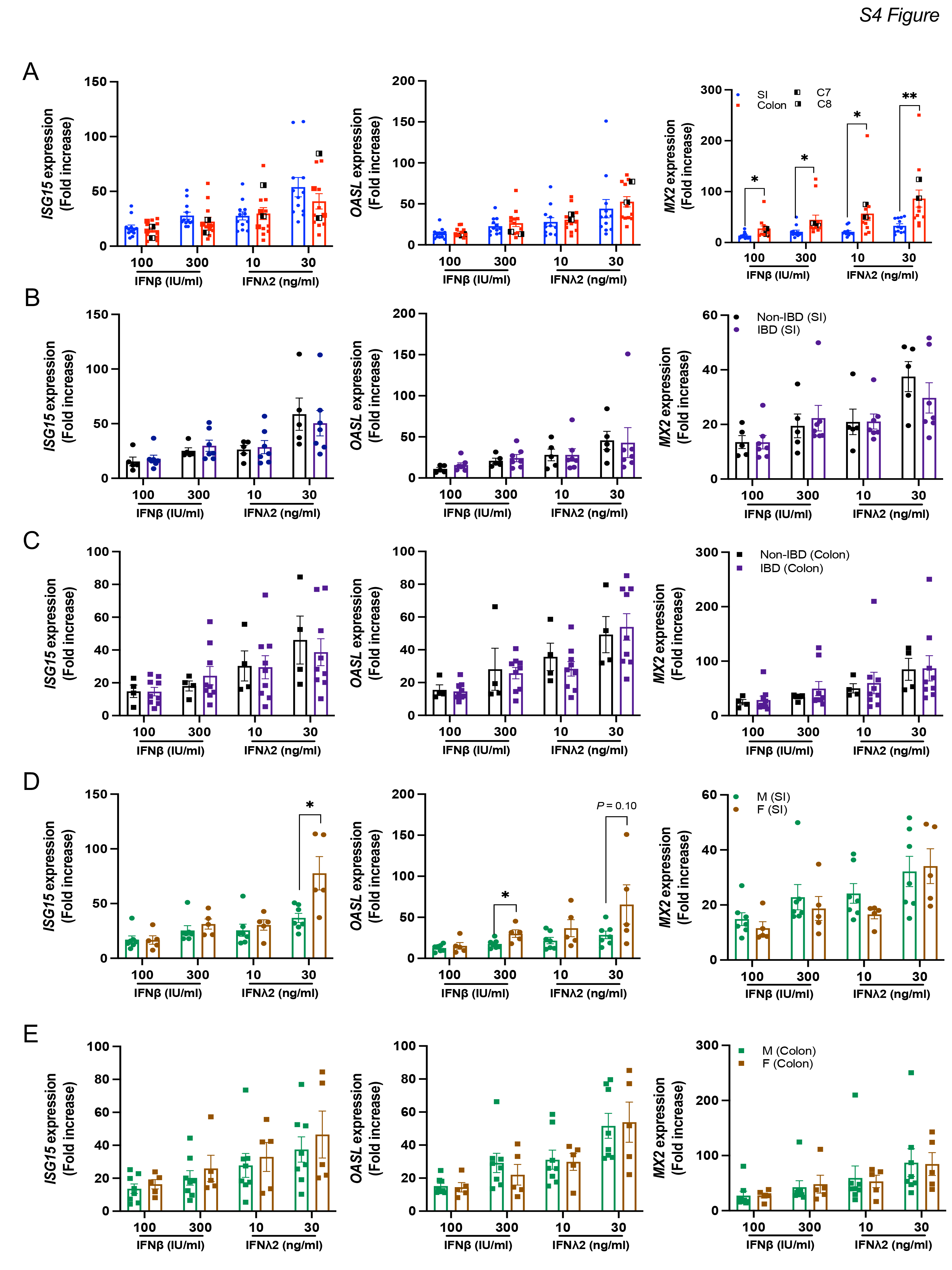

Supplement: S4 Fig — (A–E) RT-PCR data depicting fold change in ISG15, OASL, and MX2 expression in SI and colonic monolayers stimulated with IFNβ (100 or 300 IU/ml) or IFNλ2 (10 or 30 ng/ml) for 12 hours according to intestinal region (A), disease status (B and C), or sex (D and E) of donors. Each value is normalized to nonstimulated organoid lines. Data points are mean of at least 2 technical replicates of individual organoid lines. Bars represent mean ± SEM, and at least 2 independent experiments were performed. Underlying data can be found in S1 Data. P, P value. *P ≤ 0.05 and **P ≤ 0.01 by unpaired t test, 2 tailed. F, female; IFNβ, interferon beta; IFNλ2, interferon lambda 2; ISG, interferon-stimulated gene; M, male; RT-PCR, reverse transcription PCR; SI, small intestine. (TIF) [file pbio.3001592.s005.tif]

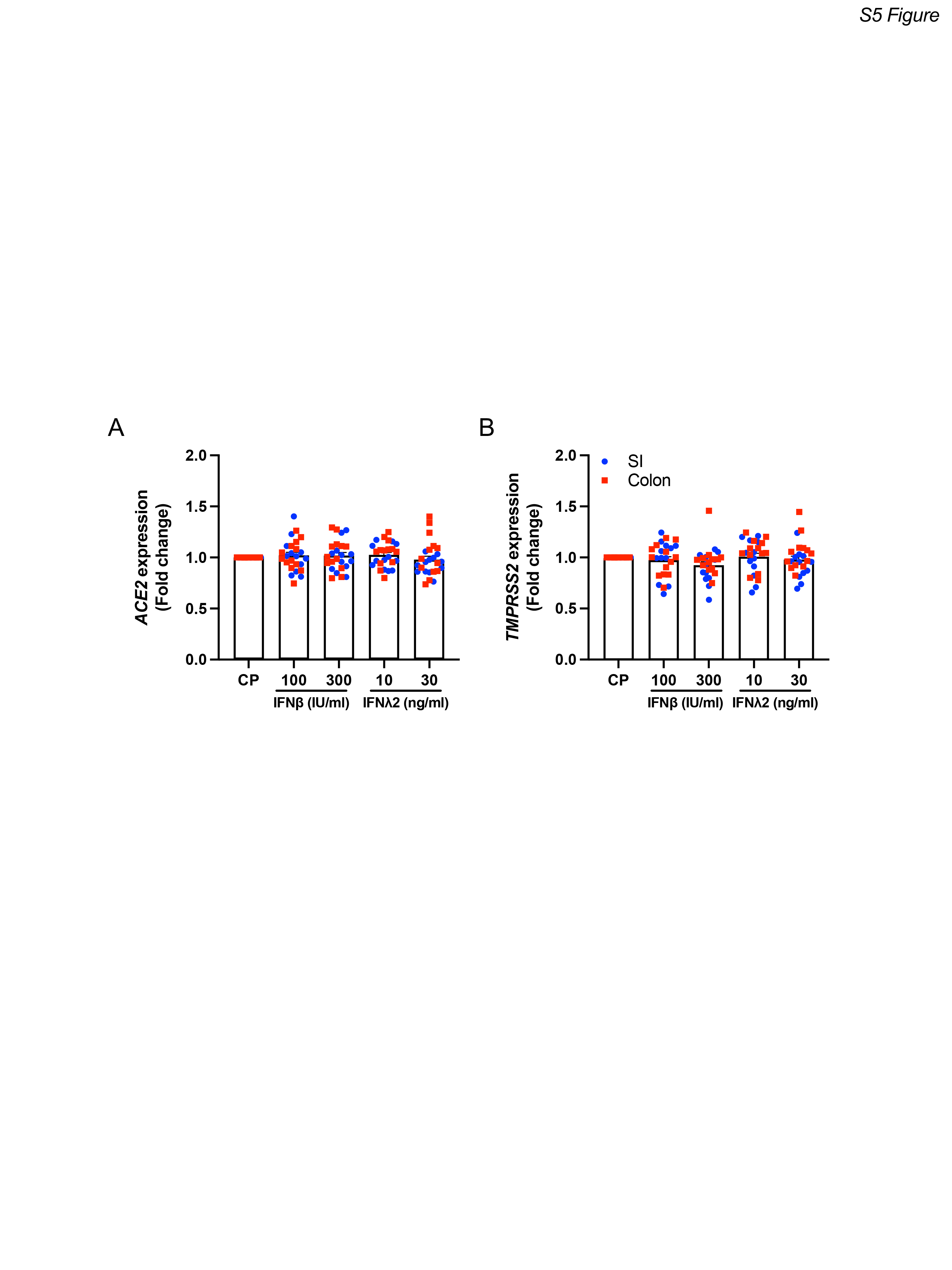

Supplement: S5 Fig — (A and B) RT-PCR depicting ACE2 (A) and TMPRSS2 (B) expression in monolayers stimulated with IFNβ (100 or 300 IU/ml) or IFNλ2 (10 or 30 ng/ml) for 12 hours. Data points are mean of at least 2 technical replicates of individual organoid lines. Bars represent mean ± SEM, and at least 2 independent experiments were performed. Underlying data can be found in S1 Data. ACE2, angiotensin I converting enzyme 2; IFNβ, interferon beta; IFNλ2, interferon lambda 2; RT-PCR, reverse transcription PCR; SI, small intestine. (TIF) [file pbio.3001592.s006.tif]

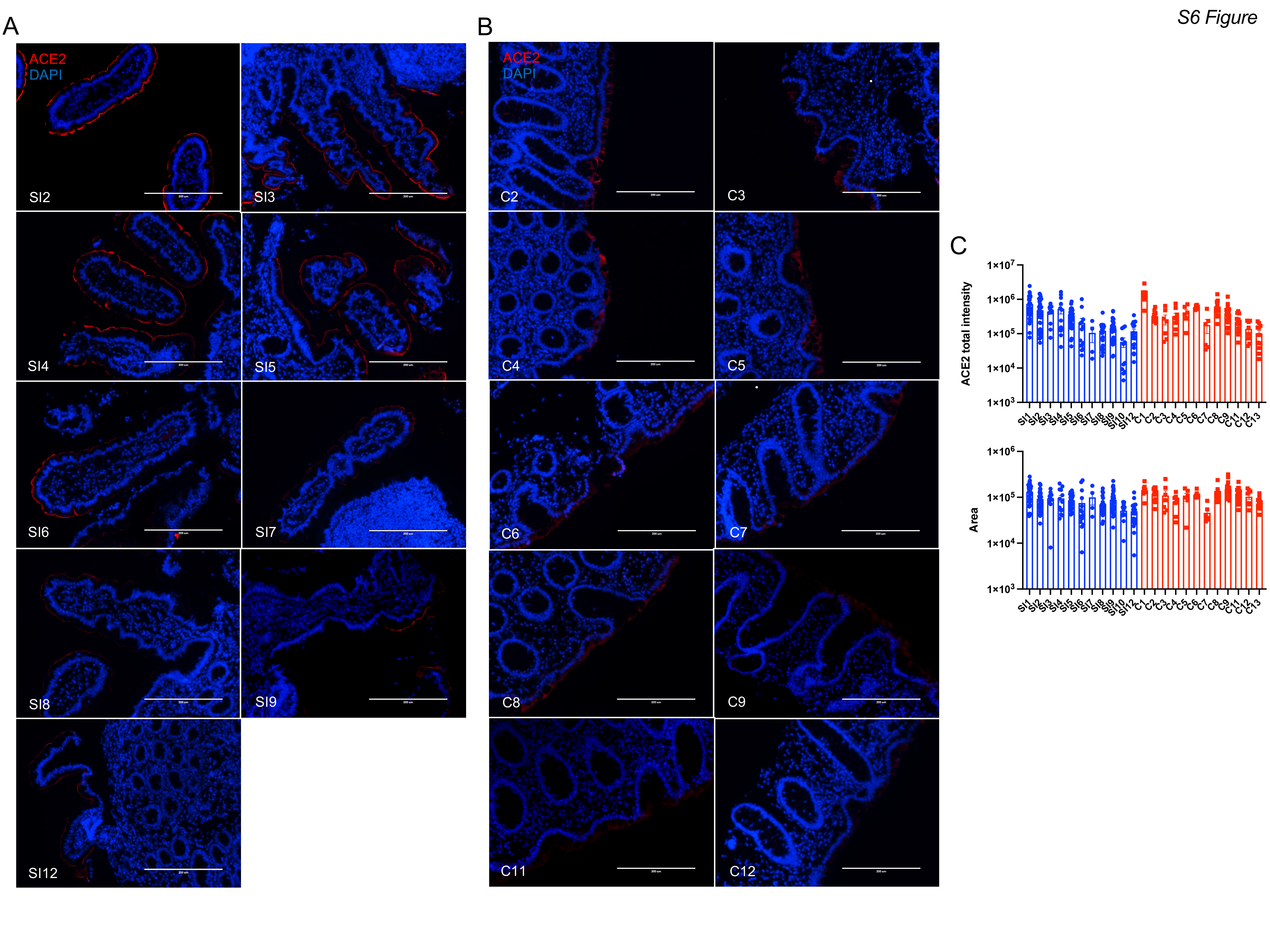

Supplement: S6 Fig — (A and B) Representative ACE2 staining images in primary tissues of terminal ileum from which SI (SI2-9 and 12, A) or colonic (C2-9, 11, and 12, B) organoid-derived monolayers were established. Images of remaining monolayers are included in main Fig 3. (C) Quantification of the total intensity of ACE2 staining and surface area in each visual field from the primary tissues from which the indicated organoid lines were established. Data points in C are the visual field. Bars in A and B: 200 μm. Bars in C represent mean ± SEM. Underlying data can be found in S1 Data. ACE2, angiotensin I converting enzyme 2; SI, small intestine. (TIF) [file pbio.3001592.s007.tif]

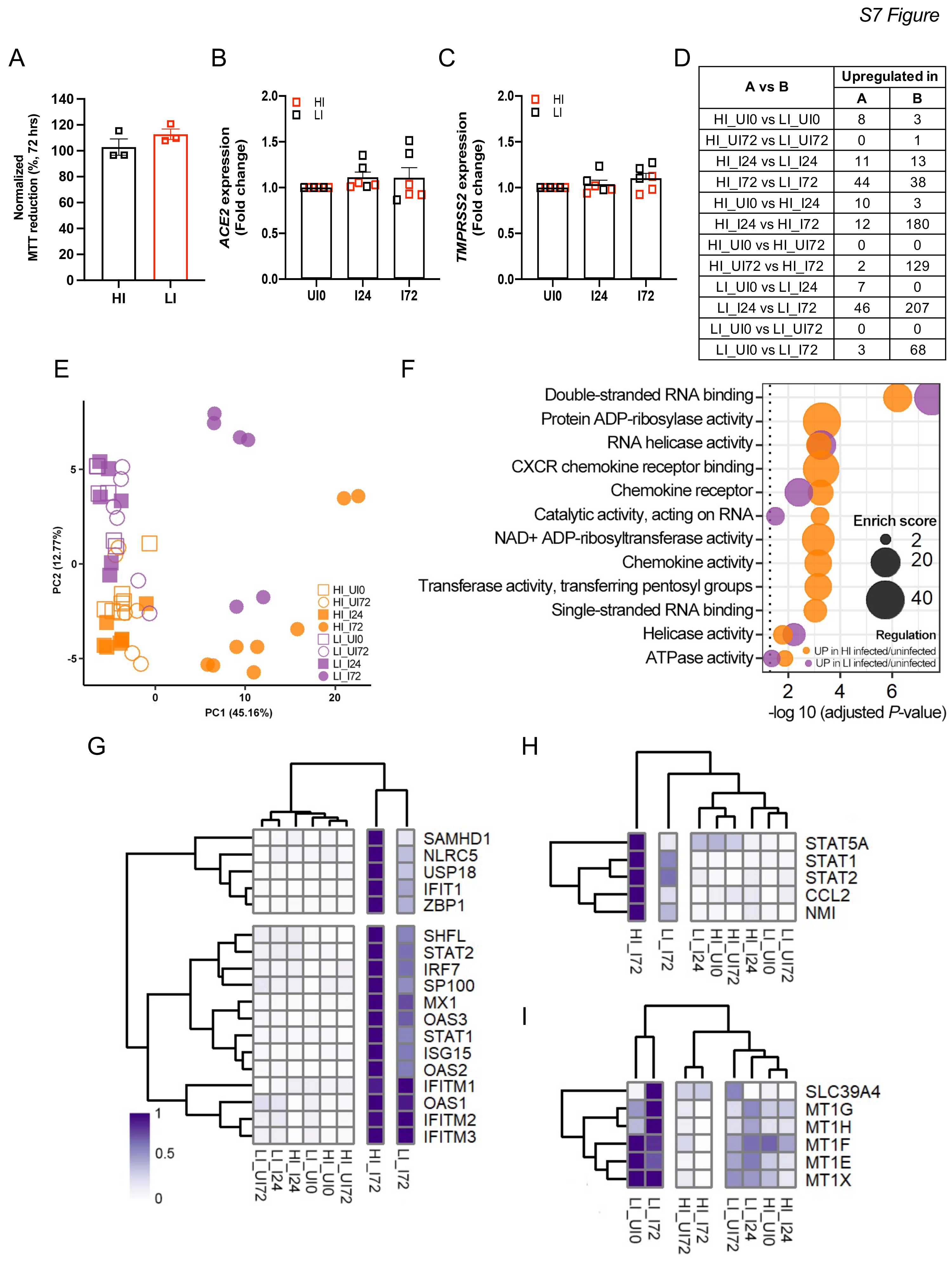

Supplement: S7 Fig — (A) Viability according to MTT reduction assay of SARS-CoV-2–infected high infection (HI; C1, C2, and C3) and low infection (LI; C8, C12, and C13) lines. (B and C) RT-PCR data depicting ACE2 (B) and TMPRSS2 (C) expression in HI and LI monolayers with or without SARS-CoV-2 infection. (D) Number of up- and down-regulated genes identified by RNA-seq analysis in the indicated pair-wise comparison of HI and LI monolayer lines at different time point postinfection. n = 3 organoid lines per group and at least 2 technical replicates per individual organoid line. (E) Unsupervised clustering based on expression of most variable genes by organoids lines and infection with SARS-CoV-2 at 24 and 72 hours. (F) Highly enriched molecular function GO terms for the DEGs in HI and LI infected with SARS-CoV-2 for 72 hours. (G and H) Heatmaps displaying normalized expression values of DEGs in HI infected/uninfected and LI infected/uninfected conditions (average fold-change >2 and adjusted P value < 0.05) annotated in GO:0060338 and GO:0034340 (G) and GO0:07259 (H). (I) Heatmap displaying normalized expression values of DEGs in LI infected/HI infected conditions (average fold-change >2 and adjusted P value < 0.05) annotated in GO:0006882. Data points in A–C are mean of at least 2 technical replicates of individual organoid lines. Bars represent mean ± SEM, and at least 2 independent experiments were performed. Underlying data can be found in S1 Data. UI0, uninfected 0 hour; UI72, uninfected 72 hours; I24, infected for 24 hours; I72, infected for 72 hours. ACE2, angiotensin I converting enzyme 2; DEG, differentially expressed gene; GO, Gene Ontology; MTT, thiazolyl blue tetrazolium bromide; RNA-seq, RNA sequencing; RT-PCR, reverse transcription PCR; SARS-CoV-2, Severe Acute Respiratory Syndrome Coronavirus 2. (TIF) [file pbio.3001592.s008.tif]

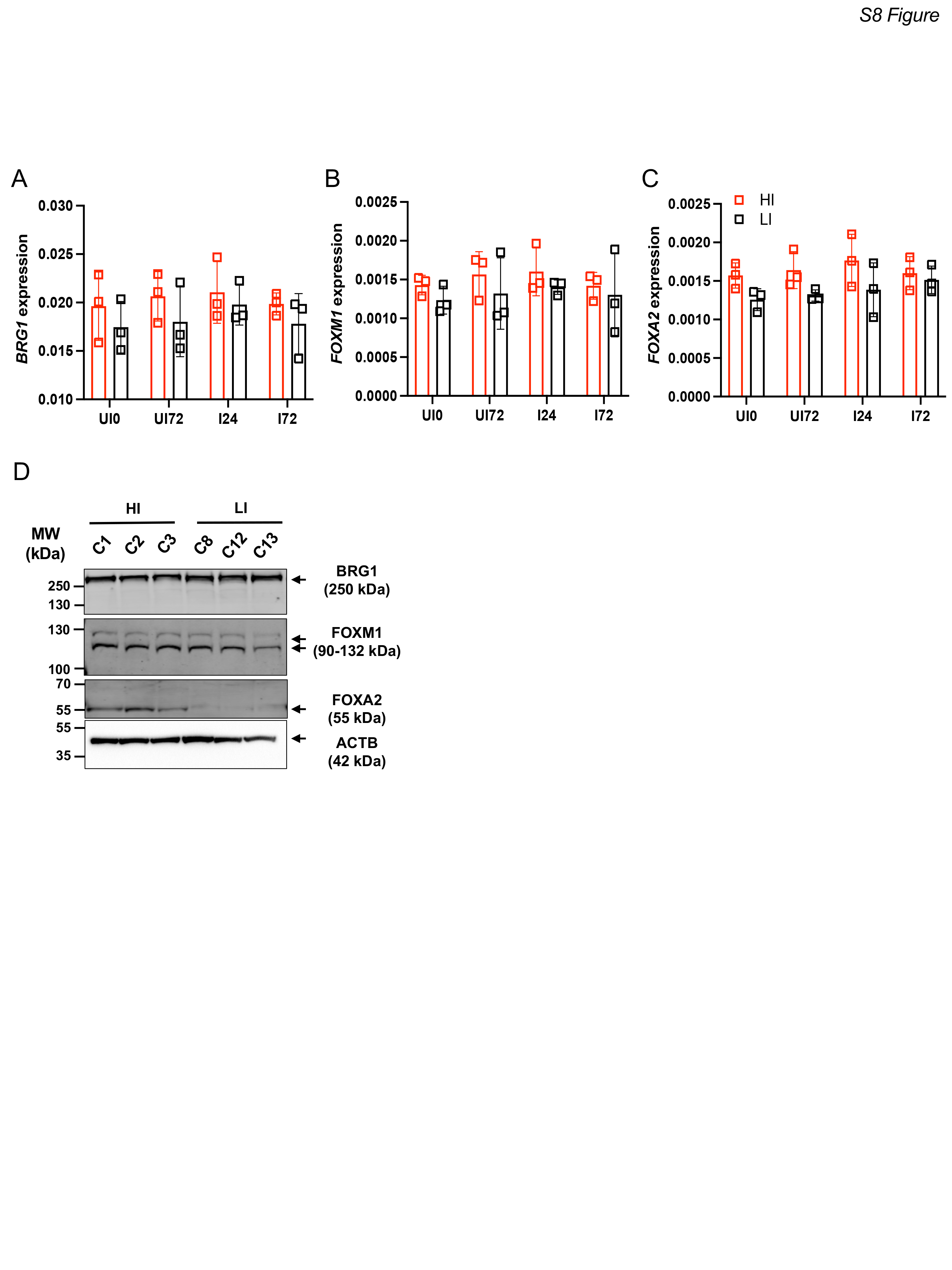

Supplement: S8 Fig — (A–C) RT-PCR analysis of BRG1 (A), FOXM1 (B), and FOXA2 (C) expression in HI and LI monolayers. (D) Western blot analysis of BRG1, FOXM1, FOXA2, and ACTB in HI and LI monolayers. Blots are representative of at least 2 independent repeats. Data points are mean of at least 2 technical replicates of individual organoid lines. Bars represent mean ± SEM, and 2 independent experiments were performed. Underlying data can be found in S1 Data. UI0, uninfected 0 hour; UI72, uninfected 72 hours; I24, infected for 24 hours; I72, infected for 72 hours. RT-PCR, reverse transcription PCR. (TIF) [file pbio.3001592.s009.tif]

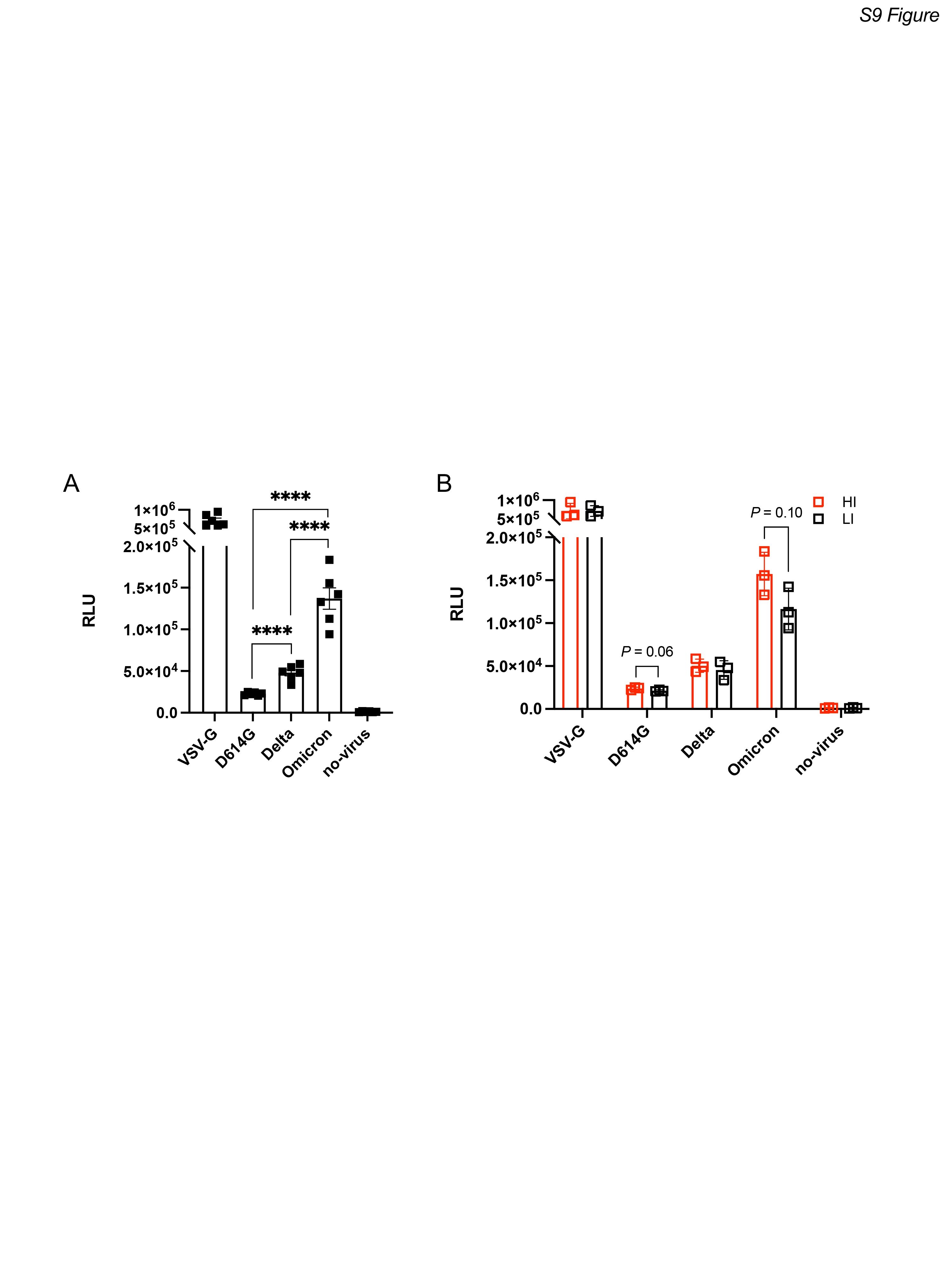

Supplement: S9 Fig — (A and B) HI and LI monolayers were infected with VSV-G or SARS-CoV-2 D614G, Delta, or Omicron S protein pseudotyped viruses at an MOI of 0.2. At 72 hours postinfection, infectivity was measured by luciferase assay. Data points are mean of at least 2 technical replicates of individual organoid lines. Bars represent mean ± SEM, and 2 independent experiments were performed. Underlying data can be found in S1 Data. P, P value. ****P ≤ 0.0001 by unpaired t test, 2 tailed. MOI, multiplicity of infection; RLU, relative luminescence unit; S, spike; SARS-CoV-2, Severe Acute Respiratory Syndrome Coronavirus 2. (TIF) [file pbio.3001592.s010.tif]

Fig 4A

**MW**  
**(kDa)**

130 -  
100 -

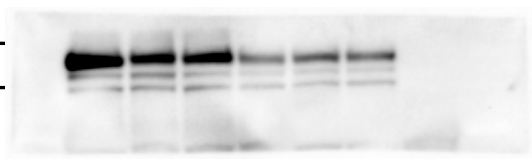

ACE2

70 -  
55 -  
35 -

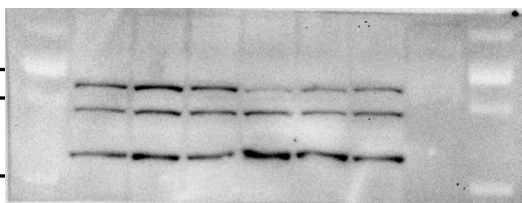

TMPRSS2

55 -  
35 -

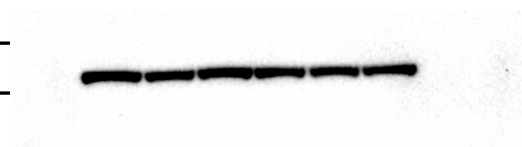

ACTB

S8D Fig

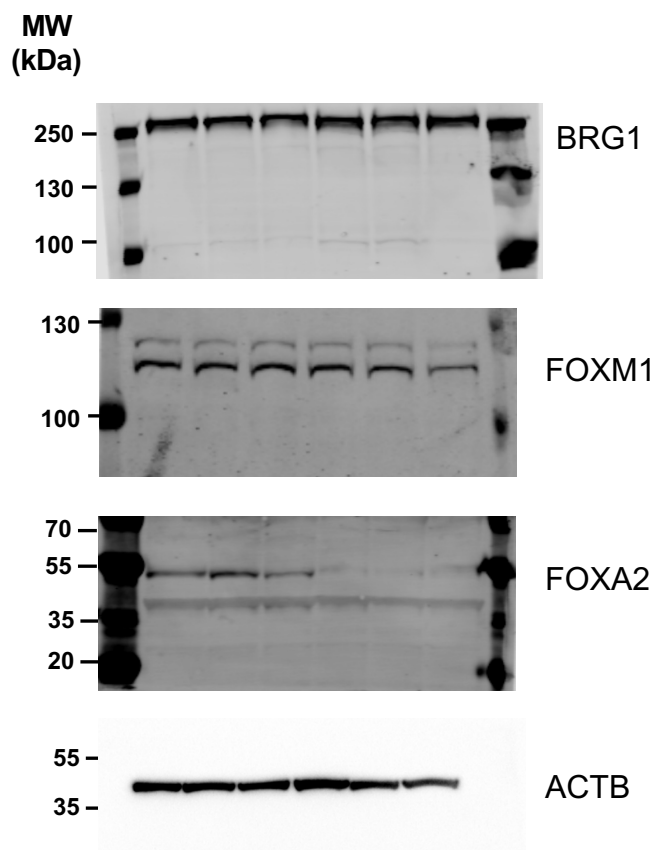

Supplement: S1 Raw Images — (PDF) [file pbio.3001592.s014.pdf]
