# Supplementary material for: Variable susceptibility of intestinal organoid–derived monolayers to SARS-CoV-2 infection
Source: PLoS Biol. 2022 Mar 31;20(3):e3001592. doi: 10.1371/journal.pbio.3001592 (PMC9004766; doi:10.1371/journal.pbio.3001592)
Supplement: S1 Table — CD, Crohn disease; F, female; IBD, inflammatory bowel disease; M, male; NA, not attempted; ND, not detected; SNP, single nucleotide polymorphism; UC, ulcerative colitis. (PDF) [file pbio.3001592.s011.pdf]

S1 Table. Subject information for human endoscopic specimens. M, male; F, female; IBD, inflammatory bowel disease; CD, Crohn's disease; UC, ulcerative colitis; SNP, single nucleotide polymorphism; ND, not detected; NA, not attempted.

Name	Tissue	Sex	Age	Disease type	Inflammation	ACE2 SNP
SI1	Terminal ileum	M	34	Non-IBD	Non-inflamed	NA
SI2	Terminal ileum	M	24	CD	Non-inflamed	NA
SI3	Terminal ileum	F	47	Non-IBD	Non-inflamed	ND
SI4	Terminal ileum	M	23	CD	Non-inflamed	ND
SI5	Terminal ileum	M	37	CD	Non-inflamed	NA
SI6	Terminal ileum	F	25	Non-IBD	Non-inflamed	NA
SI7	Terminal ileum	M	29	Non-IBD	Non-inflamed	ND
SI8	Terminal ileum	M	47	IBD	Non-inflamed	ND
SI9	Terminal ileum	F	37	CD	Non-inflamed	NA
SI10	Terminal ileum	F	69	CD	Non-inflamed	ND
SI11	Terminal ileum	F	23	Non-IBD	Non-inflamed	NA
SI12	Terminal ileum	M	39	CD	Non-inflamed	NA
C1	Ascending colon	M	34	Non-IBD	Non-inflamed	ND
C2	Ascending colon	M	25	Non-IBD	Non-inflamed	NA
C3	Ascending colon	F	55	UC	Non-inflamed	NA
C4	Ascending colon	F	27	UC	Non-inflamed	NA
C5	Ascending colon	M	29	Non-IBD	Non-inflamed	ND
C6	Ascending colon	M	33	UC	Non-inflamed	NA
C7	Ascending colon	M	33	UC	Non-inflamed	ND
C8	Ascending colon	F	23	Non-IBD	Non-inflamed	ND
C9	Ascending colon	M	24	CD	Non-inflamed	NA
C10	Ascending colon	M	37	CD	Non-inflamed	NA
C11	Ascending colon	F	37	CD	Non-inflamed	NA
C12	Ascending colon	M	21	UC	Non-inflamed	NA
C13	Ascending colon	F	20	UC	Non-inflamed	ND
