# Supplementary material for: Variable susceptibility of intestinal organoid–derived monolayers to SARS-CoV-2 infection
Source: PLoS Biol. 2022 Mar 31;20(3):e3001592. doi: 10.1371/journal.pbio.3001592 (PMC9004766; doi:10.1371/journal.pbio.3001592)
Supplement: S2 Table — P, P value; r, Pearson correlation coefficient; SI, small intestine. (PDF) [file pbio.3001592.s012.pdf]

S2 Table. Correlation between subject age and gene expression among the organoid monolayers grown in differentiation media for 7 days. r, Pearson correlation coefficient; *P*, *P*-value; SI, small intestine.

Gene expression	Age					
	SI + Colon		SI		Colon	
	r	*P*	r	*P*	r	*P*
ACE2	-0.056	0.790	-0.329	0.296	0.323	0.282
TMPRSS2	-0.122	0.562	-0.144	0.656	0.068	0.826
TMPRSS4	-0.018	0.931	0.158	0.626	0.320	0.287
APOA1	0.187	0.371	0.041	0.900	-0.235	0.439
ISG15	-0.203	0.330	-0.243	0.446	-0.145	0.636
OASL	0.004	0.985	-0.196	0.541	0.263	0.386
MX2	0.077	0.717	0.157	0.626	-0.018	0.953
