# Supplementary material for: Variable susceptibility of intestinal organoid–derived monolayers to SARS-CoV-2 infection
Source: PLoS Biol. 2022 Mar 31;20(3):e3001592. doi: 10.1371/journal.pbio.3001592 (PMC9004766; doi:10.1371/journal.pbio.3001592)
Supplement: S3 Table — P, P value; r, Pearson correlation coefficient. *P ≤ 0.05 and ***P ≤ 0.001 by simple regression analysis. IFNβ, interferon beta; IFNλ2, interferon lambda 2; PFU, plaque-forming unit; SARS-CoV-2, Severe Acute Respiratory Syndrome Coronavirus 2; SI, small intestine. (PDF) [file pbio.3001592.s013.pdf]

S3 Table. Correlation of subject age or PFU of SARS-CoV-2 with ISG induction in organoids stimulated with IFNβ (100 or 300 IU/ml) or IFNλ2 (10 or 30 ng/ml) for 12 hrs. r, Pearson correlation coefficient; *P*, *P*-value; SI, small intestine. *$P \leq 0.05$ and ***$P \leq 0.001$ by simple regression analysis.

Gene induction	Age				SARS-CoV-2 replication			
	SI		Colon		SI		Colon	
	r	*P*	r	*P*	r	*P*	r	*P*
ISG15								
100 IU/ml IFNβ	0.384	0.218	0.076	0.806	0.109	0.736	0.146	0.633
300 IU/ml IFNβ	0.311	0.326	0.041	0.894	-0.079	0.806	-0.148	0.630
10 ng/ml IFNλ2	0.433	0.160	-0.206	0.499	-0.071	0.827	-0.131	0.671
30 ng/ml IFNλ2	0.457	0.135	-0.082	0.791	-0.393	0.206	-0.028	0.927
OASL								
100 IU/ml IFNβ	0.835	*0.001****	-0.344	0.250	-0.424	0.169	-0.033	0.916
300 IU/ml IFNβ	0.668	*0.018**	0.103	0.738	-0.300	0.344	-0.096	0.755
10 ng/ml IFNλ2	0.538	0.071	-0.173	0.572	-0.404	0.193	-0.110	-0.720
30 ng/ml IFNλ2	0.623	*0.030**	0.430	0.143	-0.274	0.390	-0.163	0.596
MX2								
100 IU/ml IFNβ	-0.250	0.432	-0.102	0.741	0.308	0.331	0.006	0.985
300 IU/ml IFNβ	-0.128	0.691	-0.057	0.853	0.250	0.433	0.015	0.689
10 ng/ml IFNλ2	-0.311	0.326	-0.150	0.626	0.091	0.779	-0.194	0.526
30 ng/ml IFNλ2	0.239	0.454	-0.091	0.768	0.171	0.595	0.013	0.966
