# Supplementary material for: Variable susceptibility of intestinal organoid–derived monolayers to SARS-CoV-2 infection
Source: PLoS Biol. 2022 Mar 31;20(3):e3001592. doi: 10.1371/journal.pbio.3001592 (PMC9004766; doi:10.1371/journal.pbio.3001592)
Supplement: Attachment — Submitted filename: Jang et al_point by point response.pdf [file pbio.3001592.s015.pdf]

Summary of revisions

We sincerely thank the Editor and Reviewers for their supportive comments and helpful suggestions. We have added several new experiments including: (1) Western blot analysis of transcriptional regulators of *ACE2*, (2) infection of SARS-CoV-2 spike protein-pseudotyped reporter viruses, (3) transcript analysis of matched donor-derived small intestinal and colonic organoids, (4) MTT assay to quantify cell death in infected organoids, and (5) comparison of gene expression in 3D organoids cultured in expansion media. In addition to these experiments that bring new insight and broaden the scope of our study, we strengthened the rigor of prior experiments by: (6) adding 4 small intestinal and 3 colonic organoid lines, (7) performing ACE2 staining of primary tissue sections from the same donors corresponding to these new organoids lines, (8) using an additional housekeeping gene to normalize transcript levels detected by qPCR, (9) quantifying absolute values of *ACE2* transcripts, and (10) replacing two of the ACE2 fluorescence micrographs with better images. Finally, we have elaborated on the rationale for certain experiments and discussion topics as requested. These additions and edits are indicated in the manuscript in red. We believe these changes have improved the manuscript and addressed the critiques. Below are the point-by-point responses to each of the Reviewers' comments.

Reviewer #1

This is a well written manuscript that examines patient-derived intestinal organoids. The main conclusion is that Ace2 mRNA expression levels correlate with SARS-CoV-2 burden in human intestinal organoids that are exposed to the pathogen. In addition, the authors indicate that human intestinal organoids are useful models to study infection. While examining SARS-CoV-2 with primary human cells is certainly of broad interest, the described findings seem to lack the novel conceptual advance needed to warrant publication as a Short Report.

Response: We thank the reviewer for acknowledging that our model is of broad interest. In addition to improving the technical aspects of the manuscript, we have added a substantial amount of new data during revision, which is listed above and described in detail below. We have also tried to highlight in the revised text the ways in which our prior results, together with the new findings, represent unique contributions and advance the field. Taking into account the new data including findings with the Omicron variant spike protein, we have updated the title and abstract to clarify novelty and impact.

Some specific concerns:

1. The authors acknowledge that studies have shown that human intestinal organoids support SARS-cov2 infection and there is known variability in Ace2 expression. Their data here confirms the correlation, however does not go further or test function. Thus, the conceptual advance of this work is not clear.

Response: Previous studies have not examined interindividual variability in ACE2 levels in organoids. Also, matching ACE2 levels between organoids and tissue specimens from the same individual (biopsies taken from different sites) is another unique contribution of our study. Nevertheless, we understand the reviewer's point and considered experiments that can be performed during the revision period recommended by the journal.

To gain insight into the mechanism by which *ACE2* expression is differentially regulated, we examined the transcript and protein levels of transcriptional activators known to bind to the *ACE2* promoter, BRG1, FOXM1, and FOXA2 (1, 2). Although high infection (HI; C1, C2, and C3) and low infection (LI; C8, C12, and C13) monolayers displayed comparable levels of BRG1 and FOXM1, we observed increased protein levels of FOXA2 in HI monolayers, providing an explanation for the enhanced *ACE2* expression displayed by these monolayers (Supplemental Fig. 8D).

To increase the timeliness of our study, we tested reporter virus pseudotyped with the Omicron variant spike (S) protein (3). Interestingly, the omicron spike significantly increased virus entry into the monolayer cells. VSV-G pseudotyped control virus had the highest infectivity, which is characteristic of this virus as it uses sialic acid for entry which is present at high levels on all cells (Supplemental Fig. 9A). Despite the weaker or comparable binding affinity of Omicron S protein to ACE2 (4, 5), Omicron S protein pseudotyped virus was 2.5- and 5-fold more infectious than Delta and D614G pseudotypes, respectively (Supplemental Fig. 9A) suggesting that Omicron exploits different or additional cell entry pathways to replicate in human colonic monolayers. Consistent with our observation, a recent study deposited on biorxiv shows efficient entry of Omicron using the endosomal route (6).

Interestingly, D614G and Omicron S protein pseudotyped viruses showed 1.2- to 1.3-fold higher infectivity in HI monolayers than LI monolayers whereas the Delta S protein pseudotype displayed comparable infectivity (Supplemental Fig. 9B), indicating that the contribution of differential *ACE2* expression to viral replication is marginal. Given that the differences between

organoids are not robust for these pseudotyped viruses, we are hesitant to make strong conclusions based on this data. Still, we considered it useful to include the results and a brief discussion, and acknowledge the limitation of using pseudotyped viruses.

As mentioned above, we have updated many parts of the manuscript including the abstract to emphasize how our manuscript advances the field. We hope the reviewer considers the significant number of experiments we performed to address the other concerns from the reviewers. We believe the main conclusions are substantiated and that our manuscript is much improved.

2. Not clear on the rationale to compare gene expression between SI and colonic organoids (Fig 1F), especially since the SI and colonic organoids represent different patients and some are controls while others are derived from patients with IBD. It is not surprising that there would be extensive variability given these factors.

Response: Due to the limited amount of information in the literature regarding differences in *ACE2* expression between organoids derived from different anatomical regions (7), it was unclear to us whether small intestinal (SI) or colonic organoids would have intrinsic differences that would interfere with our ability to use them, either independently or interchangeably, as models to investigate heterogeneity. Thus, we considered it important to compare gene expression patterns both between individuals and between anatomical sites. We now clarify this point in the manuscript (line 80-83).

The reviewer makes an excellent point about comparing SI and colonic organoids derived from the same individual. We now added new data comparing *ACE2, TMPRSS2, TMPRSS4, APOA1, ISG15*, *OASL*, and *MX2* expression obtained from 5 matched pairs of SI and colonic organoids derived from the same individual. The location-dependent gene expression was reproduced in the SI and colonic-derived monolayers from the same donor (Supplemental Fig. 2A), suggesting that the gene expression pattern according to tissue location was not the results of extensive variability in the disease status of subjects.

3. Given the large initial differences in cells obtained from IBD and non-IBD patients, more in depth analyses should be included to understand basal differences in these conditions and other pathways that may correlate with SARS-cov2 infection. Figure 3D combines SI and colon in one bar for statistical comparisons. This should be justified.

Response: We agree that it would be informative to perform additional analysis of organoids obtained from IBD and non-IBD subjects to rule out differences at baseline that can be explained by tissue sampling. We found that undifferentiated 3D (3DE) organoids from IBD and non-IBD subjects displayed comparable *ACE2, TMRPSS2, TMRPSS4, APOA1, ISG15,* and *MX2* expression except the decreased *OASL* expression in colonic 3DE organoids derived from IBD patients (Supplemental Fig. 1H). Despite this lack of basal differences in these transcripts, we observed decreased *ACE2* and *APOA1* expression in differentiated colonic monolayers derived from IBD patients (Supplemental Fig. 2D and E). Thus, gene expression differences are not reflected in the initial samples and represent a property of the differentiated organoids.

We split the SI and colon into two bars and reperformed the statistical analysis in Fig 3D as suggested.

4. What is the significance of 72hours post infection for organoids? It is not clear why this is an appropriate time point to examine infection in vitro. Is there variability in other factors- cell death?

Response: This late time point was based on prior studies, which we now clarify in the revised manuscript (line 185-188). Lamers *et al* showed SARS-CoV-2 continued to display log increases in titer at 60-hrs post-infection, and only a small percentage of cells were infected at 24 hrs post-infection of human organoids (8). Furthermore, many studies suggest the interferon response to SARS-CoV-2 is delayed (8-10). Consistent with the previous literature, we observed active viral replication without corresponding changes to the transcriptional landscape in organoid-derived monolayers at 24 hrs post-infection (Fig. 4B and C and Supplemental Fig. 7D and E).

The Reviewer asks an excellent question about variability in cell death. We did not observe cytopathic effect of viral infection upon inspection by light microscopy. To more directly address this question, we now performed an MTT assay, which confirmed that cell viability remained unchanged in both HI and LI monolayers infected with SARS-CoV-2 for 72 hrs (Supplemental Fig. 7A). We include this new data in the manuscript and thank the reviewer for the suggestion.

5. In the same regard, what is the significance of the transcriptional analyses included in Figure 4. It seems expected that increased viral load would result in an increased magnitude of differences in viral-induced genes.

Response: Please see the above response for our rationale for choosing to analyze both early and late time points. To more specifically address the reviewer's comment regarding the significance of transcriptional responses at 72 hrs, we respectfully suggest that the results were not obvious before we performed the experiment. One can imagine that antiviral responses would be elevated early or at baseline prior to infection in LI monolayers, thereby explaining their enhanced resistance. Instead, the delayed increase in viral-induced genes in HI monolayers supports our model that differential susceptibility is mediated by other factors rather than antiviral immunity. We clarify this point in the revised manuscript (line 185-188).

Another reason why the 72 hr time point is interesting is that it may identify other variables or potential consequences of high versus low SARS-CoV-2 infection. For example, we observed striking induction of *MT1* genes encoding metallothionines associated with zinc and copper homeostasis in LI monolayers (Fig. 4E and H). Although not the focus of the study, we have included discussion of how elevated expression of *MT1* genes are associated with resistance to infection with hepatitis C virus and human cytomegalovirus (11-13) and zinc ion suppresses the SARS-CoV-2 replications by inhibiting its main proteases (14) (line 251-254).

<u>Reviewer #2</u>

In the manuscript entitled "Intestinal organoids expose heterogeneity in SARS-CoV-2 susceptibility", Jang and colleagues seek the inter-individual variability of ACE2 RNA and protein expression and its relationship with SARS-CoV-2 infection levels. For the study, the authors establish patient-derived organoids, followed by in vitro infection with SARS-CoV-2.

Although many figure panels are shown, the key message is straightforward. (1) Gut organoids show variability in the ACE2 expression level (2) The differential ACE2 expression in organoids reflects ACE2 expression level in the primary tissue, and (3) ACE2 expression levels of organoids show a good correlation with their SARS-CoV-2 susceptibility. Although they are interesting observations, these are not very surprising given the fact that patient-derived organoids are likely to have cellular characteristics of the specific individuals and the fact that ACE2 is the most crucial entry protein for SARS-CoV-2.

Before its publication, the reviewer thinks that a few key observations should be clarified further.

Response: We thank the reviewer for summarizing the main conclusions of our manuscript and providing the helpful suggestions listed below. We believe we have addressed all the concerns and have clarified the key observations as requested. Also, please refer to our responses to Reviewer #1, which details new content that was added during revision. Together with the experiments that address technical issues, we believe the new data and analyses significantly strengthen the manuscript.

Major comments

(1) In Fig 1, the authors explored organoids in three different environments, i.e., (1) expansion media (3DE), (2) differentiation media (3DD), and (3) monolayer. Throughout the study, the authors used (3) monolayer conditions because the condition up-regulated ACE2 levels. However, by definition, organoids are 'three-dimensional' culture models developed from a single-stem cell, and the cell cultures in the monolayer condition may not be classified as 'typical organoids', but just patient-derived primary cellular models. In such conditions, can we generalize that their findings are from "organoids"?

Response: Although other studies have used the term “organoid” to describe monolayers, we completely agree with the reviewer that this may be technically incorrect or create confusion. Therefore, we changed “monolayer organoids” to “organoid-derived 2D monolayer” in the manuscript. Note that we do not believe this affects the interpretation of our findings because past publications support the use of organoid-derived monolayers as substitutes for 3D organoids (15, 16). Also, the level of *ACE2* expression in the monolayers correlated with ACE2 protein expression in the primary tissues (Fig. 3E).

(2) In Fig 1, the authors have shown that organoids in 3DD, 3DE, and monolayer show good correlations in ACE2 RNA expression levels. However, because ACE2 expression levels (and also other genes) were measured by qPCR, (1) the ACE2 expression levels are normalized by a single housekeeping gene (GAPDH), and (2) their absolute gene expression levels cannot be understood. Transcriptome sequencing of the organoids will help clarify the issues.

Response: We agree with this concern. In the revised manuscript, we now normalize *ACE2*, *TMPRSS2*, *TMPRSS4*, *APOA1*, *ISG15*, *OASL*, and *MX2* expression in 3D organoid lines cultured

in expansion (3DE) and differentiation media (3DD) and monolayers using 2 housekeeping genes, *GAPDH* and *ACTB* encoding glyceraldehyde-3-phosphate dehydrogenase and β-actin, respectively (17). The reanalyzed qPCR data using both housekeeping genes in Figs 1, 2A and E-H, 3E, Supplemental Figs. 1, 2, 3A-D and G-I, 4, 5, 7B and C in the revised manuscript are consistent with the data normalized with *GAPDH* only. Furthermore, we quantified the copy number of *ACE2* by constructing a standard curve using an *ACE2* expressing plasmid (18) (please see the methods section). The high copy number of *ACE2* (2.9×10^5 to 1.7×10^6 transcripts/μg of RNA) in monolayers strongly correlated with *ACE2* expression and SARS-CoV-2 susceptibility (Supplemental Fig. 3D), supporting the reliable association between *ACE2* expression and SARS-CoV-2 susceptibility of monolayers. Therefore, we have improved the rigor of the transcriptional analyses in the revised manuscript and thank the reviewer for calling attention to this issue.

(3) Because the authors simultaneously compare (the colon vs the small intestine) AND (individual variations within the tissue), their messages throughout the manuscript (figures) are a bit confusing.

Response: Thank you for this advice on how to improve clarity. We moved the transcript analysis comparing monolayers derived from colonic organoids with those derived from small intestinal organoids (Figs. 1F-I and 2C in the original manuscript) to Supplemental Figs. 1I-L and 3E in the revised manuscript to improve readability.

(4) Figure 2F is one of the most important figures in the manuscript. However, excluding 1-2 outliers, the correlation does not seem to be very strong, particularly in the colon.

Response: We agree that Fig. 2F (now Fig. 2E) is one of the most important figures. As suggested by the reviewer (see Critique #6 below), we have added more samples to make sure the correlation holds true and performed all the peripheral analyses that help support our main conclusions. First, we established small intestinal and colonic 3D organoids derived from an additional 4 and 3 donors, respectively (Supplemental Table 1). The 3DE and 3DE organoids were used for transcriptional analysis, which confirmed that differences in *ISG15*, *OASL*, and *MX2* expression were conserved when we generated monolayers from these organoids (Fig. 1C and D). Interestingly, the 3DD organoids showed the highest expression of *ISG15*, *OASL*, and *MX2* among the culture conditions (Fig. 1I-K and Supplemental Fig. 1E-G).

Next, monolayers were generated and used for SARS-CoV-2 infection and transcript analyses (Figs. 1, 2A, B, and E-H, 3E, Supplemental Figs. 1I-L and 2-5). Finally, the intestinal and colonic sections from the 5 of 7 donors corresponding to the added organoid lines were used for immunofluorescence analysis (Fig. 3 and Supplemental Fig. 6). Combined results with analysis of these additional 7 lines did not change our conclusion, but yielded stronger statistical values (r and *P* values) and supported the correlation of PFU of SARS-CoV-2 with *ACE2* and *TMRPSS2* expression (Fig. 2E and F). The correlation analysis of ACE2 mean intensity with *ACE2* expression and SARS-CoV-2 levels are also more rigorous with these additional data points (Fig. 3E). Therefore, the addition of 7 lines strengthens our conclusions.

(5) The correlation between ACE2 protein levels in the primary tissues and ACE2 RNA-expression levels in the organoids is another critical part of the manuscript. The relationship was correlated in Figs 3A, 3B and 3E. Although I am not an expert in imaging, the ACE2 staining (Figs 3A, 3B) does not seem to be very convincing. Is there no ACE2 protein expression in SI7 and SI10?

Response: Thank you for pointing out the weak staining in the images. We replaced the images (SI10 and C13 in the revised manuscript) with those in which ACE2 is more visible and representative of what we observe under the microscope.

(6) In line with (5), the correlation of the central panel of Fig 3E does not seem to be very strong when we remove the single-outlier with the highest ACE2 protein expression and SARS-CoV-2 infection levels. To clarify it, validations from an additional number of individuals should be necessary

Response: As mentioned in our response to critique #4 above, we agree with this excellent suggestion and increased the number of individuals in our analysis.

Reviewer #3

This is an interesting Short Report that utilizes a biobank of human-derived intestinal organoids to investigate differences in SARS-CoV2 replication in the GI tract. The authors collected small intestine and colon biopsies from 8-10 donors, cultured organoids from these tissues under differentiated and undifferentiated states and in three-dimensions or two-dimensions, and then profiled the susceptibility of these parallel models to SARS2 infection as well as defining the host response to this infection. The authors conclude that expression of ACE2 is a primary determinant of SARS2 tropism between small intestine- and colon-derived organoids and that differential innate immune responses to infection correlate with differences in replication levels between these two systems. Although other groups have used GI-derived organoids for SARS2 studies, a clear strength of the current manuscript is the breadth of human biopsies and comparisons between differentiation states and growth conditions. The manuscript is very well-written and the conclusions are supported by rigorous parallel approaches. I have a few relatively minor points that could improve the clarity and/or conclusions of the study, which I have detailed below.

Response: We thank the reviewer for these supportive comments and for accurately summarizing the key takeaways from our manuscript. We believe we have addressed the minor critiques listed below.

1. It would seem important for the authors to mention the possible role the microbiome might play in SARS2 infection in the GI tract, which might have a significant impact on human-to-human variability and would occur independent of *ACE2* (or other host factor) expression.

Response: We agree with the reviewer that discussing the role of the microbiome would be helpful. As the reviewer mentions, several publications observed the association between gut microbiota disturbance and SARS-CoV-2 viral loads, disease susceptibility, or disease severity suggesting that heterogenous microbiome composition in the GI tract should be considered (19-22). We now discuss this possibility and include these references (line 271-275).

2. While the differential expression analysis is compelling, additional measures of differential responses that rely on IFN protein production (e.g., ELISA) would enhance the rigor and impact of this aspect of the manuscript. If the authors are unable to perform these studies, they might wish to make clearer in the manuscript that transcript levels of IFNs are not always direct correlated to protein levels, especially between IFN types.

Response: The is an excellent point. Unfortunately, we are unable to perform this experiment at this moment due to broken equipment in our BSL3 facility (we cannot remove the samples for an ELISA to be performed outside the facility). Instead, as the reviewer suggested, we highlight the importance of examining IFNs and other cytokines at the protein level for future studies that use the model described in this manuscript (line 157-160).

References

1. Pedersen KB, Chodavarapu H, Lazartigues E. Forkhead Box Transcription Factors of the FOXA Class Are Required for Basal Transcription of Angiotensin-Converting Enzyme 2. J Endocr Soc. 2017;1(4):370-84.
2. Yang J, Feng X, Zhou Q, Cheng W, Shang C, Han P, et al. Pathological Ace2-to-Ace enzyme switch in the stressed heart is transcriptionally controlled by the endothelial Brg1-FoxM1 complex. Proc Natl Acad Sci U S A. 2016;113(38):E5628-35.
3. Tada T, Fan C, Chen JS, Kaur R, Stapleford KA, Gristick H, et al. An ACE2 Microbody Containing a Single Immunoglobulin Fc Domain Is a Potent Inhibitor of SARS-CoV-2. Cell Rep. 2020;33(12):108528.
4. Wu L, Zhou L, Mo M, Liu T, Wu C, Gong C, et al. SARS-CoV-2 Omicron RBD shows weaker binding affinity than the currently dominant Delta variant to human ACE2. Signal Transduct Target Ther. 2022;7(1):8.
5. Mannar D, Saville JW, Zhu X, Srivastava SS, Berezuk AM, Tuttle KS, et al. SARS-CoV-2 Omicron Variant: ACE2 Binding, Cryo-EM Structure of Spike Protein-ACE2 Complex and Antibody Evasion. bioRxiv. 2021:2021.12.19.473380.
6. Peacock TP, Brown JC, Zhou J, Thakur N, Newman J, Kugathasan R, et al. The SARS-CoV-2 variant, Omicron, shows rapid replication in human primary nasal epithelial cultures and efficiently uses the endosomal route of entry. bioRxiv. 2022:2021.12.31.474653.
7. Zang R, Gomez Castro MF, McCune BT, Zeng Q, Rothlauf PW, Sonnek NM, et al. TMPRSS2 and TMPRSS4 promote SARS-CoV-2 infection of human small intestinal enterocytes. Sci Immunol. 2020;5(47).
8. Lamers MM, Beumer J, van der Vaart J, Knoops K, Puschhof J, Breugem TI, et al. SARS-CoV-2 productively infects human gut enterocytes. Science. 2020;369(6499):50-4.
9. Lowery SA, Sariol A, Perlman S. Innate immune and inflammatory responses to SARS-CoV-2: Implications for COVID-19. Cell Host Microbe. 2021.
10. Lei X, Dong X, Ma R, Wang W, Xiao X, Tian Z, et al. Activation and evasion of type I interferon responses by SARS-CoV-2. Nat Commun. 2020;11(1):3810.
11. Carrera G, Paternain JL, Carrere N, Folch J, Courtade-Saidi M, Orfila C, et al. Hepatic metallothionein in patients with chronic hepatitis C: relationship with severity of liver disease and response to treatment. Am J Gastroenterol. 2003;98(5):1142-9.
12. Read SA, Parnell G, Booth D, Douglas MW, George J, Ahlenstiel G. The antiviral role of zinc and metallothioneins in hepatitis C infection. J Viral Hepat. 2018;25(5):491-501.
13. Kanekiyo M, Itoh N, Kawasaki A, Tanaka J, Nakanishi T, Tanaka K. Zinc-induced activation of the human cytomegalovirus major immediate-early promoter is mediated by metallothionein and nuclear factor-kappaB. Toxicol Appl Pharmacol. 2001;173(3):146-53.
14. Panchariya L, Khan WA, Kuila S, Sonkar K, Sahoo S, Ghoshal A, et al. Zinc(2+) ion inhibits SARS-CoV-2 main protease and viral replication in vitro. Chem Commun (Camb). 2021;57(78):10083-6.
15. Wang Y, DiSalvo M, Gunasekara DB, Dutton J, Proctor A, Lebhar MS, et al. Self-renewing Monolayer of Primary Colonic or Rectal Epithelial Cells. Cell Mol Gastroenterol Hepatol. 2017;4(1):165-82 e7.
16. Nakamura T, Sato T. Advancing Intestinal Organoid Technology Toward Regenerative Medicine. Cell Mol Gastroenterol Hepatol. 2018;5(1):51-60.
17. Vandesompele J, De Preter K, Pattyn F, Poppe B, Van Roy N, De Paepe A, et al. Accurate normalization of real-time quantitative RT-PCR data by geometric averaging of multiple internal control genes. Genome Biol. 2002;3(7):RESEARCH0034.
18. Li W, Moore MJ, Vasilieva N, Sui J, Wong SK, Berne MA, et al. Angiotensin-converting enzyme 2 is a functional receptor for the SARS coronavirus. Nature. 2003;426(6965):450-4.
19. Wu Y, Cheng X, Jiang G, Tang H, Ming S, Tang L, et al. Altered oral and gut microbiota and its association with SARS-CoV-2 viral load in COVID-19 patients during hospitalization. NPJ Biofilms Microbiomes. 2021;7(1):61.
20. Yeoh YK, Zuo T, Lui GC, Zhang F, Liu Q, Li AY, et al. Gut microbiota composition reflects disease severity and dysfunctional immune responses in patients with COVID-19. Gut. 2021;70(4):698-706.
21. Venzon M, Bernard L, Klein J, Axelrad JE, Hussey GA, Sullivan AP, et al. Gut microbiome dysbiosis during COVID-19 is associated with increased risk for bacteremia and microbial translocation. bioRxiv. 20

21:2021.07.15.452246.
22. Zhang F, Wan Y, Zuo T, Yeoh YK, Liu Q, Zhang L, et al. Prolonged Impairment of Short-Chain Fatty Acid and L-Isoleucine Biosynthesis in Gut Microbiome in Patients With COVID-19. Gastroenterology. 2021.
